# Quality of Life of Persons Injured on 9/11: Qualitative Analysis from the World Trade Center Health Registry

**DOI:** 10.1371/currents.dis.7c70f66c1e6c5f41b43c797cb2a04793

**Published:** 2016-10-27

**Authors:** Lisa M. Gargano, Robyn R. Gershon, Robert M. Brackbill

**Affiliations:** New York City Department of Health and Mental Hygiene, World Trade Center Health Registry, Division of Epidemiology, Long Island City, New York, USA; Philip R. Lee Institute for Health Policy Studies and Department of Epidemiology and Biostatistics, School of Medicine, University of California, San Francisco, California, USA; New York City Department of Health, World Trade Center Health Registry, New York, New York, USA

## Abstract

Introduction: A number of studies published by the World Trade Center Health Registry (Registry) document the prevalence of injuries sustained by victims of the World Trade Center Disaster (WTCD) on 9/11. Injury occurrence during or in the immediate aftermath of this event has been shown to be a risk factor for long-term adverse physical and mental health status. More recent reports of ongoing physical health and mental health problems and overall poor quality of life among survivors led us to undertake this qualitative study to explore the long-term impact of having both disaster-related injuries and peri-event traumatic exposure on quality of life in disaster survivors.

Methods: Semi-structured, in-depth individual telephone interviews were conducted with 33 Registry enrollees who reported being injured on 9/11/01. Topics included: extent and circumstance of the injury(ies), description of medical treatment for injury, current health and functional status, and lifestyle changes resulting from the WTCD. The interviews were recorded, transcribed, and inductively open-coded for thematic analysis.

Results: Six themes emerged with respect to long term recovery and quality of life: concurrent experience of injury with exposure to peri-event traumatic exposure (e.g., witnessing death or destruction, perceived life threat, etc.); sub-optimal quality and timeliness of short- and long-term medical care for the injury reported and mental health care; poor ongoing health status, functional limitations, and disabilities; adverse impact on lifestyle; lack of social support; and adverse economic impact. Many study participants, especially those reporting more serious injuries, also reported self-imposed social isolation, an inability to participate in or take enjoyment from previously enjoyable leisure and social activities and greatly diminished overall quality of life.

Discussion: This study provided unique insight into the long-term impact of disasters on survivors. Long after physical injuries have healed, some injured disaster survivors report having serious health and mental health problems, economic problems due to loss of livelihood, limited sources of social support, and profound social isolation. Strategies for addressing the long-term health problems of disaster survivors are needed in order to support recovery.

## Introduction

A large number of studies have shown that victims of natural and human-made disasters experience both adverse physical and mental health effects, with survivors especially at risk of post-traumatic stress disorder (PTSD) and depression.^1^^,^ 2 ^,^ 3 ^,^ 4 ^,^ 5 The World Trade Center Health Registry (Registry) was developed to understand the short, medium, and long-term public health impacts of the attacks on people exposed to the World Trade Center (WTC) disaster on September 11, 2001 (9/11). The Registry enrolled more than 70,000 people who were members of populations at risk, including: those in the vicinity of the attacks in lower Manhattan on 9/11/01 either in buildings (including the WTC Towers) or passing through the area; residents who lived in the immediate area surrounding the WTC site; rescue, recovery and ancillary workers working at the site on 9/11 or on later clean-up and recovery operations; and children who were enrolled in schools in the vicinity of the WTC.^1^^,^ 6

A study published by the Registry found that over 9,000 (13%) enrollees reported being injured on 9/11.6 The most common injury reported was a sprain or strain.^6^ Some groups were more likely to be more seriously injured; for instance, while 3,672 (44%) of Registry enrollee survivors were injured, 474 (13%) reported an injury involving a fracture or dislocation, burn, or head injury.^7^ These and other Registry studies have also found injury to be a significant risk factor for mental health disorders, including a two-fold higher likelihood of PTSD, after controlling for other risk factors.^7^^,^ 8 The occurrence of any injury was also found to be independently associated with new onset heart disease post-9/11.^8^ In addition to well-documented on-going health and mental health problems noted by Registry enrollees who were injured, there have also been numerous anecdotal reports of poor quality of life reported by survivors. Whether this is related to injuries sustained on 9/11 or to peri-traumatic exposure to the disaster, or both, is an open question, as it is hard to disentangle the long-term effects of injuries from long-term effects of the traumatic exposure.

After the attacks, the New York State Office of Mental Health collaborated with New York City and county mental health departments to address mental health needs using two related but distinct response strategies.^9^ The first, named Project Liberty, aimed at the general population consisted of public education concerning traumatic stress reactions and appropriate coping strategies, outreach to all affected communities, and short-term supportive counseling for anyone affected by September 11th. The second response strategy was aimed at a subset of the affected population: individuals whose traumatic symptoms persisted and were of sufficient severity to meet diagnostic criteria for PTSD and/or other mental disorder. From mid-October 2001 through March 2002, Project Liberty staff provided over 42,000 service encounters, representing service to over 91,000 unique individuals. An evaluation of Project Liberty showed that counselors were able to identify which individuals might require more intensive mental health treatment. Overall, about 9% of individuals encountered through Project Liberty were referred for mental health treatment. These individuals experienced about twice as many traumatic symptoms as those not referred, and rates of referral were higher for highly traumatized groups such as families of the deceased and WTC evacuees.^9^

Information on disaster-related factors that may influence long-term quality of life of survivors can inform the development of strategies to ensure the best possible outcome and recovery for seriously injured disaster survivors. The goals of this qualitative study were to 1) fill in the gaps in knowledge concerning the circumstances surrounding the injuries, 2) determine the short and long term mental and physical health effects of being injured, and 3) assess the social and economic impacts of being injured.

## Methods

A semi-structured interview script, which was informed by a conceptual model of long-term health impacts in disaster survivors, was used to conduct the interviews. Telephone interviews were conducted on 33 eligible Registry enrollees between March and June 2015 (83% of those contacted by phone agreed to be interviewed). Up to 50 interviews were planned, but saturation^10^ was reached with 33 interviews and therefore, in order to minimize respondent burden, no further recruitment was conducted. These interviewees met the study criteria eligibility: a) completion of registry survey waves 1, 2, and 3; b) report of sustaining any one or more of five types of injuries on 9/11 (sprain/strain, cut/laceration, burn, fracture, or head injury) on the Registry’s Wave 1 (2002-2003) interview; c) report of being present south of Chambers Street on the morning of 9/11; d) aged 17 years or older on 9/11; e) English language preference; f) and current address in U.S. We maximized representation of persons who either reported multiple types of injuries or more severe types of injuries by including those with head/concussion, burns, or bone fractures or dislocation injuries, while also including persons with less severe types of injuries, such as sprain/strain or lacerations. For the first 10 interviews, we did not recruit persons with a history of probable PTSD, which is defined as attaining a score of 44 or greater on the PCL-17^1^^,^ 11 in order to assess the degree of distress during the interview that might occur. In subsequent batches we recruited persons with probable PTSD at Waves 1 or 2 and then Wave 3. Recruiters and the interviewer were blinded to the PTSD scores of all participants in order to minimize the risk of interviewer bias.

Potential study participants were first sent an invitation letter with a description of the study, its purpose and the benefits and risks of participation. Registry recruitment staff called all potential participants to schedule an interview and to obtain verbal consent prior to conducting the interview. Participants were sent a $50 gift card as compensation for their time.

Each interview lasted approximately one hour. The interviewer’s guide was developed by all authors with input from other Registry staff. Table 1 displays topics and sample questions from the guide. All interviews were recorded with enrollee permission and transcribed verbatim by a professional transcriptionist. This study was approved by the institutional review board of the NYC Department of Health and Mental Hygiene.

**Ethics Statement**

This study was approved by the institutional review board of the NYC Department of Health and Mental Hygiene.

**Figure:**
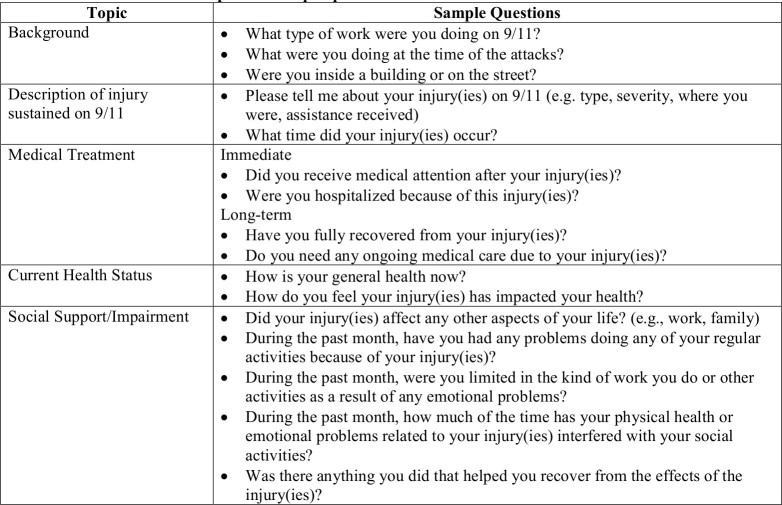
**Table 1. **Interview topic and sample questions

**Data Analysis**

Using a pre-set list of major themes that were based on the conceptual models embedded in the interview script, two members of the research team read and coded the first five transcripts of the recorded data. The analysis was conducted using the block of data for each of the major categories as described in the interview guide. The researchers then met to achieve consensus on the major themes and sub-themes, and to identify and discuss any new themes that emerged from the data. At this stage, the researchers also agreed upon the terminology that would be used for the themes. For example, originally, one researcher listed “Changes in Work Status” as a separate theme, the two researchers agreed to fold this into the “Lifestyle Impacts” theme. One researcher initially referred to any new health problems as “New Health Problems,” which was then changed to “Current Health Status, Functional Physical Status Impairments and Disability.” Once the new terminology was in place, the two researchers then read the next 5 transcripts to ensure that they were coding using the same terminology and pre-set themes similarly. After thorough discussion of the identified themes by all three researchers, further adjustments to terminology were again made at this point to keep the themes to a manageable number (resulting in the final six themes identified). Disagreements about themes were discussed and resolved among coders until 100% agreement on themes was achieved. Two researchers then reread all 33 transcripts and coded them separately. They then met to compare their results and at this point the third researcher assessed the coding and determined an interrater reliability of 0.97.

The authors used thematic analysis^12^ as the analytic framework to identify themes relevant to participants’ post-9/11 health and mental health (short- and long-term), health care utilization, social support, and everyday functioning. Coders were unaware of the PTSD scores during this analysis phase. The analysis included line-by-line coding of statements and responses by two independent researchers (LMG and RG).^13^ The researchers reviewed the codes and evaluated their meaning. The coded data were organized to identify themes. These themes were cross-referenced among the coders and percent agreement was determined.

## Results

**Participant characteristics**

Demographic characteristics of the participants are described in Table 2. Over half (57.6%) were male and 48.5% were age 25-44 years on 9/11. The largest proportion (75.8%) of participants was white and 12.1% were Hispanic. Almost half (45.5%) had a college or post-graduate degree. Over half (51.7%) reported an annual household income of over $75,000 in 2002. Almost 40% participated in rescue and recovery work on 9/11. Almost half (48.5%) did not screen positive for probable PTSD on any of the 3 waves, 27.3% had probable PTSD at Wave 1 and/or Wave 2, and 24.2% had probable PTSD at Wave 3. Three-quarters of participants had ‘more’ severe injuries, of these, 69.7% had two or more injuries, and 24.2% were categorized as having ‘less’ severe injuries.

**Figure:**
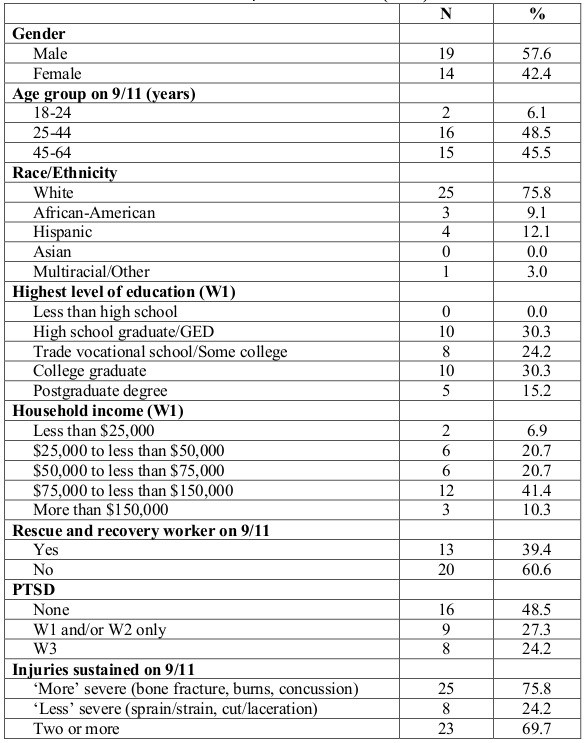
**Table 2. **Characteristics of participants interviewed (n=33)

**Thematic analysis**

Thematic analysis yielded six themes related to exposure to WTCD and current quality of life: (1) concurrent peri-event injuries and traumatic exposures on 9/11; (2) timeliness of short- and long-term medical care for injury and mental health care; (3) current health status, functional limitations, and disabilities; (4) lifestyle impact; (5) availability of social support; and (6) economic impact. Interrater reliability for each theme was good (kappa = 0.97). Themes are presented below, with representative quotations selected to highlight key results.

**Figure:**
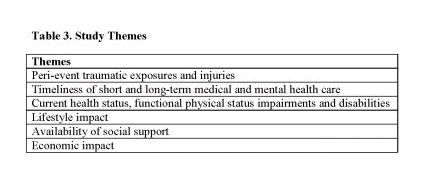
**Table 3. **Study Themes

**Peri-event traumatic exposures and injuries**

The first awareness of the event varied greatly and was dependent upon the participant’s location when the event first began at 8:46am. Some participants were already at their desks, located in either of the towers or in surrounding buildings. Others were at the street level and were about to enter one of the seven WTC complex buildings or other nearby buildings. Unless the participant had a direct view of an airplane making impact, making sense of the situation was initially, at least, virtually impossible. Even participants who heard the first explosion as the plane made impact and reported seeing debris from their office windows could not understand what had just happened. Almost no one initially understood the gravity of the situation or the precariousness of their own personal safety. In these first few minutes, there was a sense of confusion and uncertainty regarding what had happened and how they should respond.

Sensory exposure was common. Participants reported seeing and hearing the planes impact the buildings, and seeing the collapse of either the WTC Tower One (WTC 1) or WTC Tower Two (WTC 2), or both. Participants saw people on high floors in WTC 1 waving tablecloths and then jumping out of windows. Participants saw and felt debris from the towers and the impact of the dust clouds. Participants smelled smoke and other odors of building materials caught up in the dust cloud. Participants heard screaming of others when they were in their work area, in the stairwells, and on the street.

Participants experienced a wide range of injuries, including, most commonly, eye injury, foot injuries, skin burns, head injuries, contusions, sprains, strains, and broken bones. Several participants experienced injuries almost immediately after the first impact, generally because of resultant damage to their work area (falling ceilings and walls). Others who were at street level were injured by falling debris. Still others were injured in the lobby of WTC 1 when a fire ball exploded out of the bank of elevators. Other victims were not injured until they began to evacuate, either from WTC 1 or WTC 2 or from other WTC complex buildings or buildings in the vicinity. Several participants were injured later on, during and immediately after the collapse of WTC 2, at 9:59am, or later still, when WTC 1 collapsed at 10:28am. Participants who were not injured either immediately or shortly after the attacks reported that their injuries occurred during their sometimes extended journey towards home or to another destination.

The participant’s location at the start of the attacks at 8:46am also affected the intensity of their peri-event traumatic exposure. As described by one participant, *“…then I just heard this horrible, indescribable sound. It sounded like the biggest thing you ever heard was tearing through a jungle. And I turned around and I saw it [the plane impacting WTC 1].*”

The initial thought of many participants was that they were going to die, as reported by one participant, *“I said, listen God, make it quick. I don’t want to suffer.”* Many participants reported feeling the experience was ‘other worldly’, as one participant noted, *“I experienced an alien abduction feeling.”* For many participants, a sense of disbelief was common; they tried to make sense of what they were seeing. Dust, darkness, smoke and confusion were commonly reported. One participant, a first responder heading to the complex, reported that when he emerged from the Holland Tunnel, the scene was* “a nuclear winter; I just remember the dust and smoke, and the confusion.”* There was also a sense of disbelief for some that they were still alive, as one participant, buried in the rubble after the collapse of WTC 2 stated, *“I realized, I’m alive.”* For some participants, the reality of what they had experienced did not become clear for quite some time after the event. One participant, who woke up later in an intensive care unit, reported, *“[The nurse] looked at me and said, ‘you know, you are the luckiest person in the entire world.’”*

Part of the traumatic exposure was related to witnessing people getting injured or killed, in some cases, quite near them. It was clear from the participants’ reports that the traumatic event was a combination of both witnessing the unfolding events, including escaping past corpses in the tower lobby and on the street (remains of jumpers from high floors), as well as getting caught up and injured in the event itself. For instance, several participants were traumatized during their efforts to run away from the collapsing buildings amid the flying debris and resultant dust clouds, not only because they were injured during these efforts, but because they were caught up in stampeding crowds of people who were similarly trying to flee. As noted by one participant, as WTC 1 started to collapse, *“It was every man for himself. We just started running.”* Another survivor reported a similar situation when WTC 2 collapsed,* “I started running and there were just hundreds of people running. And somebody just completely knocked the wind out of me and knocked me across the street itself. And I really thought that I was just going to die there.”*

Although first responders were on the scene early to direct people out and away from the buildings, many interviewees reported that once WTC 2 collapsed, conditions on the ground rapidly deteriorated, making escape especially traumatic. Visibility became exceptionally poor; as noted by one participant,* “It was the darkest- it was darker than nighttime. It was indescribable really.”* In the darkness, people became disoriented, and several of them experienced injuries at this point.

As the evacuation inside of the buildings proceeded, conditions in the stairwells degraded. At one point, in WTC 2, shortly before the collapse of the building, several participants reported chaos in the stairwell; large cracks appeared in the stairwell walls, all the emergency lights went out, water from broken standpipes was filling in and reached up to the knees of some people, and people began to panic and run down the stairs. Several participants also noted that it was at this point or shortly after they exited the stairwells, they were now without their shoes and experienced foot injuries.

The collapse of the towers was harrowing for several participants as the force of the implosion caused them to be thrown. As noted by one survivor,* “[I was] about 30 feet I'd say from the North Tower when it collapsed. And I was blown about 40 feet in the air. I smacked into a cement wall. I was able to crawl into a room in WTC Tower 6.”* Other survivors similarly reported getting thrown in the air as they were hit by flying debris. As noted by one first responder running away from the collapsing WTC 1,* “I got maybe about two blocks, 2 1/2 blocks up, and I got hit on the head and I went flying and I think I fell into what was an excavation pit…I was buried over with concrete and debris.”* Other participants also reported getting buried under debris, as noted by one survivor, *“Since we were so close, you know the force just moved me – I don't know – like 20 feet or however. It definitely blew me a distance. And we were all flattened out. Everybody who was in the area. And then there was that huge gray-black cloud. You couldn't see any sunlight – like just a tad, just a smidgen. And after we were all knocked down ….somebody was on top of me because of the weight of everything coming down. That's when I fractured my rib. And I had my leg pulled up behind me and I had an injury to my knee.”*

Participants reported getting buried under debris and losing consciousness as the towers collapsed. As one survivor of WTC 1 collapse stated, *“I ran towards building five [WTC Tower 5]. That's the shortest entrance you can run to in order to get out of the staircase when the building [started to] collapse. I blacked out and the next thing I know I was on a stretcher and getting medical attention.”*

**Timeliness of short and long-term medical and mental health care**

Immediate medical care for injuries fell into three categories – none, immediate, and delayed. Most, though not all, of those who did not seek any medical care (either immediate or delayed) had ‘less’ severe injuries, mostly cuts and scrapes, foot injuries, and eye irritation. Those with ‘more’ severe injuries received either immediate or delayed medical care for their injuries, with the majority generally receiving care by the end of the day, although some waited days to weeks before receiving care. The reasons for delaying care included,* “did not realize how badly I was injured”, “could not/would not leave my home/apartment,”* and,* “wanted to be alone/not think about the event.”*

Many of the more severely injured people first had to be evacuated from the immediate vicinity of the WTC to another location. In some cases, this took quite an extended period of time because they were first evacuated by boat to a triage site, usually in Staten Island or New Jersey, and from there they were then taken by bus, ambulance, or private car to a nearby hospital. Even though many of these more severely injured people were in great pain, they self- evacuated or were assisted by ‘Good Samaritans’ to these more distant locations - without benefit of immediate first aid care (by Emergency Medical Services) or pain medications. As one participant reported, *"They got me into triage- I remember screaming, somebody give me some morphine."* When they finally arrived at the distant hospital, hours after they had initially sustained their injuries, many of these participants reported that they basically were ‘in shock,’ with many completely unaware of how they had even gotten there. In addition to receiving care and treatment of their injuries, many of the participants who presented to either local or more distant hospitals reported that in addition to treatment of their injuries, they also received pulmonary therapy treatments to help them breathe. No one in our sample reported that the hospital provider recommended that they seek follow-up psychological care, although several were urged to see other medical specialists and/or their family provider.

Some study participants who were first responders reported that even though they had been badly injured that day, they still refused medical care. Most felt they were needed on the job and did not want to stop working, others were clearly in shock (they afterward said they could not remember details of how they had continued to perform their duties), some had received head injuries (and may also have been in shock), and others felt it was unseemly to complain about their injuries when so many fellow rescuers had been killed. Other (non-responder) participants declined any offers of care that day as they wanted to reach their own provider near where they lived. However, several did not get to see their own provider for many days, and when they did, the provider also did not discuss the potential need for follow-up mental health care.

Among those with more severe injuries, long-term care and continued health problems were commonly reported. Almost all participants who had sustained serious injuries spoke of the continued need for physical therapy as well as continuing assessment and treatment by medical specialists on a regular basis, such as orthopedists and pulmonologists. Several participants mentioned that long after they had sustained their injuries, they required surgery, sometimes multiple surgeries. Several mentioned that additional future surgeries would be needed to repair ongoing problems, most typically on the knees, shoulders, and back. A few of these participants also discussed being in chronic pain and having a constant need for pain medications. Several participants also mentioned self-medicating after 9/11, including the use of herbal preparations. Persons in the interviews often mentioned heavy alcohol consumption for a short time after 9/11. Participants who reported current self-medicating all had current probable PTSD. One participant discussed problems sleeping because of nightmares and when asked how they managed this, they replied, *“I take some NyQuil© and a glass of wine.”*

**Current health status, functional physical status impairments and disability**

Long term disability in those who had suffered orthopedic injuries was not uncommon. In some cases, participants stated that they were partially or fully disabled as a result of their injuries. Some participants stated that they are now disfigured, as noted one participant who had sustained serious burns, and who did not like to go out in public because, as he said, *“Kids say, mommy look at that man's hands.”*

Some participants described mobility impairments that required the use of wheelchairs, walkers, or canes. Others had chronic injury-related health problems (such as loss of range of motion in injured limbs or chronic pain). One participant who reported broken bones in his fingers, reported now having arthritis in those fingers, saying* "[It’s] so bad that I can't even barely tie my shoes or anything like that."* One participant who had suffered severe burns spoke of loss of dexterity in hands and loss of sensitivity in finger tips. Physical symptoms and diseases described less frequently included hearing loss, dermatological conditions, autoimmune diseases, and cancer. When discussing his current health and hearing loss, one participant said, *“[The doctor] said ‘your hearing loss is remarkably advanced for a man your age.’”*

Several participants stated that their injuries resulted in a diminution in their ability to physically perform their normal activities of daily living, such as bathing, dressing, cooking, cleaning, etc. As noted one participant, *“I’ve got to accept the fact that I can’t do nothing like I used to.”* Many seriously injured participants who had previously been very physically active (playing sports, going to the gym, participating in outdoor activities) now reported that they could no longer do these things, as one person noted, *"Life has certainly changed."*

In addition to the long-term effect of physical injuries, almost all of the participants reported a variety of respiratory symptoms and conditions, including asthma, reactive airway disease, sleep apnea, bronchitis, chronic pneumonia, and lung problems. These respiratory problems also negatively impacted their current functional status. One participant said* “I can't do any [type of] work for any length of time without literally sitting down and trying to catch my breath.”* Similarly, another participant stated *“Even now, when I start to do anything strenuous, bingo, forget it, it takes me a while to recover from it”* Many participants also reported sinus and throat problems such as allergies, throat irritation, and acid reflux/gastroesophageal reflux diseases (GERD). After one participant had an endoscopy, he said he was told that *“…it looked like I had swallowed some red hot coals.”*

In addition to physical ailments, many of the participants described a range of ongoing mental health symptoms related to the event, including anxiety, depression, panic attacks, and sleep disturbances. Several had been diagnosed with PTSD, anxiety disorder and/or depression by a clinician. Serious mental health problems were noted, as recalled by one person who said, *"[after 911], I would look at people and I would see their skeleton."* When asked how often this happened, the response was,* “All the time.”* For most participants reporting mental health symptoms, these generally became evident shortly after the WTC event, although others reported that symptoms did not manifest until weeks or months later. Several participants reported that their mental health symptoms have persisted for years. Anxiety symptoms included reluctance to fly, fear of being in crowds or public spaces, other phobias, and nervousness. Some participants reported that they were generally now more “emotional” than they were before 9/11. One participant stated, *"I'm very emotional now. Yeah. I am, [I] mean anything, it has me crying."* While another said, *"Some days I'm fine and then there's other days where I get totally upset."* Participants also mentioned having sleep problems, restlessness, nightmares, and insomnia. One participant described *"My wife was telling me that half a dozen times I'd be crying in my sleep, yelling in my sleep. She said I'm worried about you. You're not right, you're not yourself. And I knew I wasn't myself. I didn't want my kids going out, I didn't want them going anywhere."* Some, but not all, of the participants with these types of symptoms eventually received mental health services such as counseling therapy and prescription medications. Several participants reported that they had moved away from New York City to avoid any reminders of the event, but that even after the relocation, their symptoms persisted.

**Lifestyle impact**

Almost all participants reported major changes in their lifestyle after 9/11. Some lifestyle changes were related to the loss of their jobs, and in other cases, changes resulted because of their injuries and subsequent disabilities, as illustrated by this quote,* “I left… [or rather] I would say, that I was forced to take early (disability) retirement as a result of it. My wife works now and I just take care of the kids.”*

Several participants reported that they now had little or no enjoyment in life and that they no longer had any interest in participating in activities they had previously (prior to 9/11) found enjoyable. Examples of these activities included socializing with friends and family, working out, playing sports, outdoor activities with family, working around the house and garden, and playing with their children. One participant said, *“I'm always one to say that I would never talk about any injuries because 2753 people would've traded places with me that day. So I don't really talk about [my] injuries. But it really did change my life. Especially having a two month old at the time. You [normally] want to be with them all the time and do things. So I've been a lot less active with my younger kid than I was with my now 20-year-old.”*

Others spoke of missing social or cultural activities because they avoided going into Manhattan or taking the subway or flying on airplanes. A participant commented: *“If I have to go to the city for a function, I’m a wreck.”* When another participant was probed further about their ability to take the subway they said, *“I can, but it's not something that I want to do and it's not something – I'll stay up all night before- if I know I'm going to go to the museum or take the train…”*

Other participants described more extreme social isolation and a near total lack of contact with people and society (e.g., not wanting to leave their home, avoiding going out with friends). One participant commented: *“I didn't want to go out, I didn't want to see anybody.”* When asked about leisure activities and going out with friends, another participant simply said,* “No, that [all] stopped.”*

**Availability of social support**

Some study participants discussed different sources of support that helped them, not only recover from the effects of their injuries, but to deal with the totality of the events of 9/11. The most commonly cited source of support was family. One participant said,* “I have to say that I think the fact that I have a very good home life has the biggest impact. I mean my wife is – she's constantly caring for me. Outside of our children I'm her biggest priority. And so I would have to equate her support as being my biggest coping mechanism.”* When discussing the importance of the support he received from his wife and daughter, another participant said, *“… because if it weren’t for them I don’t know what would’ve happened to me.”* Additional sources of social support mentioned by participants were religion, therapy, friends, and volunteer activities.

Some participants however reported a dearth of social support. They felt that the only people who truly understood them were other 9/11 survivors. One participant described it as, *“You talk to the people you were with that know what you’ve been through. It’s very difficult to talk to other people about it.”* This lack of ability to discuss feelings and emotions related to 9/11, coupled with wanting to be isolated from everyone led to estrangement in families.

**Economic impact**

Participants reported a variety of economic consequences, and most were negative. Some economic impacts were directly due to an injury sustained on 9/11, including the inability to work and the increased costs associated with medical care. Many of the participants with more severe injuries, as well as those with subsequent health issues, had to retire with disability benefits or take early retirement. One participant on permanent disability said, *“Economically I’m in a hard spot. I had to take a home equity loan on my house to make ends meet.”*

Other economic impacts were related more generally to the events of 9/11. Several participants reported that they had lost their job due to company downsizing or going out of business after 9/11. One participant mentioned the financial impact of having to move out of the area, *"I mean the whole event was a severe economic impact because I was basically in a rent-controlled apartment in Battery Park and I had to move. So when I had to move, my rent tripled."*

## Discussion

As has been previously reported,^14^ many participants reported delay in seeking health care services after the event. Oftentimes, the delay was quite extended, even when the participant had experienced relatively serious injuries. This was especially common among first responders who tended to dismiss their own injuries so that they could return to duty as soon as possible. They also did not want to “make a big deal of the injury,” as so many fellow responders had been killed. A similar sentiment was also voiced by non-responders; they felt guilty that they had survived and did not want to acknowledge injuries in the face of so many fatalities. To address this issue, recommendations regarding care for all acutely exposed survivors (with or without obvious injuries) should include medical evaluation and treatment, as needed, at the earliest possible time following mass casualty events. The medical evaluation should also include assessment for acute stress response, with follow-up scheduled so that physical health status could be monitored and to identify psychological problems that may manifest at a later time (weeks or months after the event). This follow-up visit would also allow for referral for any specialized medical or psychological treatment. Programs like Project Liberty, started in the weeks after 9/11, should be implemented to provide free public educational and crisis counseling services. This large-scale public health intervention aimed at ameliorating the traumatic stress experienced by tens of thousands of New Yorkers in the disaster area.^9^ Individuals varied widely in the severity of experienced trauma and associated traumatic reactions and individuals with the most severe reactions were referred to longer-term mental health treatment services.^9^ Primary care providers now furnish over half of mental health treatment in this country and about 25% of all primary care recipients have diagnosable mental disorders.^15^ In disaster exposed populations the need for psychological care may rapidly overwhelm the available mental health services, and primary care providers could be a source of mental health treatment after a disaster. In addition, this approach would also allow for an integrated mental health services with physical healthcare in the same location, an approach that has been shown to be highly effective.^16^

As shown in previous studies on 9/11 survivors enrolled in the World Trade Center Health Registry, long-term physical and mental health problems were common.^1^^,^ 5 ^,^ 8 ^,^ 17 ^,^ 18 The risk factors for long-term disability found in this study have also been reported in other research on 9/11 survivors, namely severity of injury, intensity of peri-event traumatic exposure, and lack of timely and effective mental health care.^18^^,^ 19 ^,^ 20 A novel finding in our study was the extent of extreme social isolation in some participants. The upheaval this event caused in their lives led some survivors to become very socially withdrawn, which then in turn led to further adverse effects on their overall quality of life. In many cases, social isolation in many participants resulted in them being home bound, manifested especially in fearfulness of being out in public or in public spaces. Many participants no longer engaged in activities and/or social events that had previously been enjoyable for them. Extreme fear about leaving the home led to estrangement from their family members and friends. Several became very anxious about their children and spouses leaving the home- especially if they were going to be in crowded places, which also caused problems within the family. Sleep disturbances and chronic pain led to self-medication, which also led many to withdraw from social contact.

It was not surprising to find that participants stressed the importance of emotional and social support from friends and family, as high social support and social integration have been shown to decrease the odds of 9/11-related PTSD.^21^^,^ 22 An important finding is that some participants said they were only able to talk about their 9/11-experiences with others who had also been there during the attacks. This may indicate a need for group therapy sessions or support groups after a disaster, for both the individual and potentially their family. In cases where the participant did eventually enter mental health care treatment, treatment was often reported to be efficacious, especially if they were able to form a strong therapeutic bond with their therapist. The need for not only effective evidence-based individual mental health care for disaster survivors, but for their families as well, is an important consideration raised by these study findings.

Early retirement or disability retirement was commonly reported, many participants were left without important benefits of work. A recent study of WTCHR enrollees found that workers with chronic conditions were more likely to experience early retirement and job loss, and the association was stronger in the presence of PTSD comorbidity.^23^ A qualitative study of injured survivors of The Station nightclub fire in Rhode Island also found that participants commented on themes of financial stress as well as disruption to their daily lives, such as inability to work.^24^ Beyond the issue of loss of income, which was very common in our sample, loss of employment eliminated opportunities for workplace socialization and access to employee assistance programs, leading further to social isolation. Moreover, because many firms went out of business, employees no longer even stayed in touch with one another. This was true even for first responders who felt disconnected from their former friends, as they no longer felt part of ‘the team.’ In some cases though, retired first responders formed new bonds with other WTCD retirees.

We found that even among those who did not have current PTSD, there was a lack of recovery and diminished quality of life reported. There are a number of possible reasons for this observation. One explanation might be a relatively new phenomenon referred to as “moral injury”.^25^ There are similarities between reports of ongoing symptoms and sense of isolation in these participants with those reported by returning war veterans. In our study, participants frequently witnessed suffering, horrific and undignified death, and fear for their lives, and the symptoms commonly associated with moral injury (guilt, remorse, and demoralization)^25^ were also frequently reported by our study participants. Additional studies to assess this phenomenon in disaster survivors, especially survivors of terrorist or mass-shooting atrocities are warranted. Another potential explanation could be that participants have some form of survivor’s guilt. Survivor’s guilt is a mental condition that occurs when a person perceives themselves to have done wrong by surviving a traumatic event when others did not.^26^ It may be found among survivors of combat, natural disasters, and epidemics.^26^ In the Diagnostic and Statistical Manual of Mental Disorders V (DSM-V), it is considered an associated descriptive feature of PTSD, described as a persistent negative trauma-related emotion.^27^ Survivor guilt has also been implicated in the genesis of clinical depression. Although major clinical depression and PTSD are often comorbid diagnoses, guilt may be present or absent in both.^28^

On the other hand, just how participants who demonstrated resiliency were able to do so may be explained by recent work by Richardson (2015) on the role of ‘making sense’ in disaster recovery.^29^ Programs, such as the docent program at the WTC Tribute Center as described by Richardson, have been shown to help support recovery by allowing survivors to make better sense of their trauma through sharing for posterity their own experiences on that day. This gave them both a sense of purpose and a sense of pride that they could help others.^30^

There is a growing interest in post-traumatic resilience and post-traumatic growth, which can result from devastating, catastrophic events.^31^ Though there may be a tendency to look to survivors of a traumatic event as victims of a tragedy, future research into long-term outcomes should focus on factors contributing to survivor resiliency and positive outcomes in the face of tragedy.

**Limitations**

This study focused solely on those injured on 9/11 and thus results reported here may not be representative of other non-inured 9/11 disaster survivors. With only 33 participants, this was a small sample, although we did reach thematic saturation. There was also the risk of recall bias, however, we did have data from 2002-2003 on injuries reported at that time and we also framed the questions on their current recovery and everyday functioning. Also, there is the possibility of self-report bias; participants may have provided responses that they felt were socially acceptable. In spite of the potential limitations inherent in the design and sampling, this exploratory study was appropriate for this stage of inquiry and provided unanticipated avenues for further research.

## Conclusions

In this study we were able to identify serious long-term health and mental health effects related to the WTCD. Among those injured, quality of life was seriously and negatively affected. Public health has a responsibility to aid survivors and community members both immediately following a disaster event and, importantly, in the long-term. Because both natural and human-caused disasters are increasing in frequency and severity, communities and government agencies should prepare now with respect to the provision of short and long-term assessment and treatment for victims of these types of events. The full functioning and participation of all members of the community is essential for community resiliency and recovery following disasters.

## Data Availability Statement

Due to ethical restrictions, the qualitative data in the study is only available upon request. For further information regarding data availability please contact Lisa Gargano: lgargano1@health.nyc.gov.

## Corresponding Author

Lisa Gargano: lgargano1@health.nyc.gov

## Competing Interests

The authors have declared that no competing interests exist.
